# Repetitive Sequence Stability in Embryonic Stem Cells

**DOI:** 10.3390/ijms25168819

**Published:** 2024-08-13

**Authors:** Guang Shi, Qianwen Pang, Zhancheng Lin, Xinyi Zhang, Kaimeng Huang

**Affiliations:** 1 MOE Key Laboratory of Gene Function and Regulation, Guangzhou Key Laboratory of Healthy Aging Research and SYSU-BCM Joint Research Center, School of Life Sciences, Sun Yat-sen University, Guangzhou 510275, China; pangxw3@mail2.sysu.edu.cn (Q.P.); lamchinch@mail2.sysu.edu.cn (Z.L.); zhangxy@mail2.sysu.edu.cn (X.Z.); 2 Division of Radiation and Genome Stability, Department of Radiation Oncology, Dana-Farber Cancer Institute, Harvard Medical School, Boston, MA 02215, USA; kaimeng_huang@dfci.harvard.edu; 3 Broad Institute of Harvard and MIT, Cambridge, MA 02142, USA

**Keywords:** repetitive sequences, epigenetic regulation, DNA damage, embryonic stem cells

## Abstract

Repetitive sequences play an indispensable role in gene expression, transcriptional regulation, and chromosome arrangements through trans and cis regulation. In this review, focusing on recent advances, we summarize the epigenetic regulatory mechanisms of repetitive sequences in embryonic stem cells. We aim to bridge the knowledge gap by discussing DNA damage repair pathway choices on repetitive sequences and summarizing the significance of chromatin organization on repetitive sequences in response to DNA damage. By consolidating these insights, we underscore the critical relationship between the stability of repetitive sequences and early embryonic development, seeking to provide a deeper understanding of repetitive sequence stability and setting the stage for further research and potential therapeutic strategies in developmental biology and regenerative medicine.

## 1. Introduction

Throughout the evolution from prokaryotic to eukaryotic organisms, the proportion of repetitive sequences in the genome has gradually increased. In particular, about half of the mammalian genome consists of repetitive sequences. Compared to 4% of coding genes in the human genome, these repetitive sequences were initially considered “junk DNA”, but actually play an important role in evolution, inheritance, and variation [1,2]. They are also particularly vital in early embryonic development and disease progression, influencing gene expression, transcriptional regulation, and chromatin structure through trans- and cis-regulatory roles.

Due to the properties of repetitive sequences, they can form alternative non-B DNA structures in the process of DNA replication and transcription, resulting in fork stalling and collapse, repetitive sequence contraction and expansion, and genome instability [3]. In addition, retrotransposons are abundant in the mammalian genome. The activation of intact retrotransposons can integrate into the genome through a “copy and paste” mechanism, inducing genomic instability [4,5,6]. In fact, as a major source of endogenous DNA damage, the accumulation of DNA damage on repetitive sequences is harmful to embryonic stem cells (ESCs) [3,7,8]. Therefore, it is crucial to protect repetitive sequences from DNA damage in the process of DNA replication and transcription [9,10,11], and the expression levels of retrotransposons need to be precisely regulated during early embryonic development. This review focuses on the recent research advancements, summarizing the epigenetic regulation mechanisms that contribute to the stability of repetitive sequences in ESCs. We also discuss new insights into the connection between repetitive sequence damage and repair, as well as higher-order chromatin organization on repetitive sequences in response to DNA damage.

## 2. Epigenetic Regulation of Repetitive Sequences in ESCs

**Figure 1:**
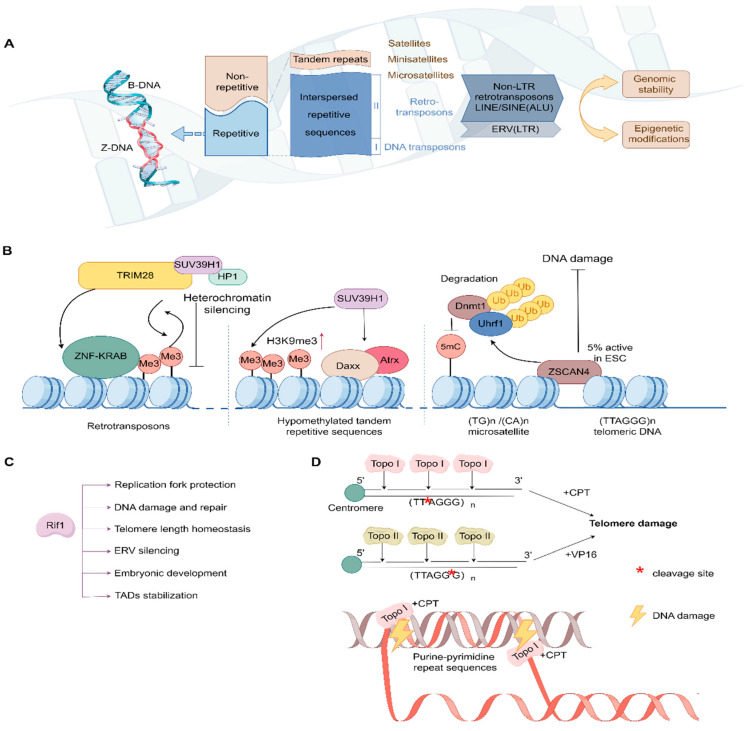
Classification and stability regulation mechanisms of repetitive sequences. (**A**) Classification of repetitive DNA sequences. Repetitive DNA sequences are divided into two classes: interspersed repetitive sequences and clustered tandem repetitive sequences. Tandem repetitive sequences are classified into satellites, minisatellites, and microsatellites. Interspersed repetitive sequences are mainly copies of transposable elements (TEs), including DNA transposons and retrotransposons. Among them, retrotransposons are mainly divided into two superfamilies: non-LTR retrotransposons and endogenous retroviruses. (**B**) Epigenetic mechanisms regulate repetitive DNA sequences (TEs and tandem repetitive sequences). (**C**) Rif1 is involved in DNA replication fork protection, DNA damage repair, telomere length homeostasis, ERV silencing, embryonic development, and TAD stabilization. (**D**) DNA topoisomerases and their special inhibitors mediate DNA cleavage and DNA damage on the telomeric DNA and purine–pyrimidine repeat sequences.

Approximately 50% of the human genome is composed of repetitive sequences [2,6,12]. In terms of the distribution of repetitive sequences within the genome, there are two main classes: interspersed repetitive sequences (~48%) and clustered tandem repetitive sequences (~3%) (Figure 1A). Interspersed repetitive sequences are mainly copies of transposable elements (TEs), including DNA transposons and retrotransposons, with the ability to transpose and insert at other places in the genome [6]. According to the lengths of the basic units, tandem repetitive sequences are classified into satellites (>100 bp per repeat), minisatellites (10–100 bp per repeat), and microsatellites (also called short tandem repeats, <10 bp per repeat) [13]. Over the course of the evolution from eukaryotes to mammals, both the proportion and the number of repetitive sequences in the genome are gradually increased. Extensive research has demonstrated that repetitive sequences are not “junk DNA”, and mainly affect evolution, inheritance, and variation [14,15]. In terms of molecular regulation, they also play an indispensable role in gene expression, transcriptional regulation, and chromatin structure [6]. Nevertheless, the role and function of repetitive sequences still need further exploration.

TEs in humans are classified into class I (retrotransposons, ~43%) and class II (DNA transposons, ~5%) [6]. Among them, retrotransposons need to be transcribed to RNA, reverse-transcribed to cDNA, and then integrated into the genome via a “copy and paste” mechanism. According to their structure and transposon patterns, retrotransposons are mainly divided into two superfamilies: long and short interspersed nuclear elements (LINEs, ~21%; SINE or ALU in humans, ~13%) without long terminal repeat sequences (also called non-LTR retrotransposons), and endogenous retroviruses (ERVs) with long terminal repeat sequences (ERVs/LTRs, ~9%) [6] (Figure 1A). LINEs, as autonomous retrotransposons, can encode reverse transcriptase and proteins for transposition [16,17], whereas ERVs, SINE, and ALU have non-autonomous elements and depend on long interspersed element-1 (LINE-1)-encoded proteins for transposition [18,19].

Retrotransposons are dynamic and stage-specific; are active in early embryos, germlines, and pluripotent stem cells [20,21,22,23]; and are important for embryonic and germline development. In particular, retrotransposons as naive state markers, help ESCs and induced pluripotent stem cells (iPSCs) to maintain pluripotency, and even promote reprogramming from pluripotency cells to totipotent-like cells [24,25,26,27,28,29,30]. On the contrary, active retrotransposons are described as inducing genomic instability, disrupting regulatory elements, causing mutations, and driving genome evolution [31,32,33]. In order to repress retrotransposon transposition and maintain genomic stability, retrotransposons in ESCs are regulated by repressive epigenetic modifications (e.g., DNA methylation, H3K9me3) and certain protein factors [2,34,35,36,37,38], always accompanied with heterochromatin silencing, which is correlated with the dynamic expression level of retrotransposons. DNA methylation enzymes (Dnmt3a, Dnmt3b, or Dnmt1) targeting retrotransposons cause DNA hypermethylation and repress their transcription. The loss of Dnmt3a, Dnmt3b, and Dnmt1 cause methylation reduction in mESCs. The repression of LINEs is mainly mediated by Dnmt3a/3b, whereas ERVs are repressed by Dnmt1 [39]. After the deletion of the H3K9 methyltransferase Setdb1, hundreds of ERVs lose H3K9me3 and are derepressed [38]. These results demonstrate that retrotransposons have two sides and their expression level needs to be precisely regulated by epigenetic modifications.

Recent research indicates that repetitive DNA binding proteins play an important role in the epigenetic regulation of repetitive sequences, mainly through recruiting repressive epigenetic factors (Figure 1B). To suppress retrotransposon transcription, ESCs employ the KRAB-ZFP transcription factor family to bind these elements, then recruit histone methylase SUV39H1, heterochromatin protein HP1, and TRIM28 to establish repressive heterochromatin [35,37,40]. Specifically, KRAB-zinc finger protein Zfp819 interacts with TRIM28 and inhibit repetitive sequence transcription in mESCs [41]. In Zfp819 knockdown mESCs, the expression of ERVs was significantly upregulated [41]. Moreover, it was reported that the DNA-binding protein ZFP57 targeted retrotransposon elements to maintain DNA methylation and repressive histone marks during embryonic development [42].

In certain developmental contexts where the genomic DNA is hypomethylated, such as inner cell mass in vivo, repetitive sequences are not dysregulated, suggesting additional regulation mechanisms to maintain repetitive sequence silencing (Figure 1B). It was reported that some factors bind and protect hypomethylated tandem repetitive sequences. For instance, Death-associated protein (Daxx)/alpha-thalassemia X-linked syndrome protein (Atrx) protected hypomethylated tandem repetitive element in the ground pluripotency state, showing increased binding of Daxx/Atrx and H3K9me3 levels [43]. Zinc finger and SCAN domain containing 4 (Zscan4), which was crucial for two-cell and four-cell stage transition during early embryonic development, bond to (TTAGGG)n telomeric DNA and (TG)n/(CA)n microsatellite DNA, protecting mouse two-cell embryos from DNA damage [44]. Zscan4 also facilitates telomere elongation by inducing global DNA demethylation through the downregulation of Uhrf1 and Dnmt1, major components of DNA methylation maintenance machinery [45].

## 3. Proteins Involved in DNA Replication Are Likely to Protect Repetitive Sequences from DNA Damage

Compared to single-copy DNA sequences in the genome, repetitive sequences are more susceptible to accumulating DNA damage during DNA replication and transcription. Due to the properties of repetitive sequences, they can form alternative non-B DNA structures, such as left-handed Z-DNA, cruciform DNA, triplex-helix DNA, and hairpin [46]. These structures may interfere with DNA replication and transcription, resulting in fork stalling and collapse, repeat contraction or expansion, and genome instability [8,47,48]. In this event, structure-forming repeats may also affect DNA damage repair. Given the relatively longer S phase in ESCs [49,50,51], there is enough time for DNA repair to prevent DNA damage accumulation. In fact, the accumulation of DNA damage triggered by DNA replication arrest is one of the main reasons for failure in preimplantation development. Therefore, protecting repetitive sequences from DNA damage during DNA replication and transcription is crucial in early embryonic development.

Recent research has shown that the proteins involved in DNA replication and genomic stability are likely to regulate repetitive sequences and play important roles in embryonic development (Figure 1C,D). For instance, RAP1 interacting factor 1 (Rif1), a multiple functional protein conserved from yeast to mammals, plays critical roles in replication fork protection, DNA damage and repair, telomere length homeostasis, ERV silencing, and the stabilization of topologically associated domains (TADs) [52,53,54,55,56,57] (Figure 1C). Rif1 is highly expressed in mouse embryos and mouse ESCs (mESCs), and its loss promotes mESC differentiation [58]. In Rif1 knockout mESCs, the transcripts of endogenous retrovirus MERVL and noncoding RNA of major satellite repeats are increased, and these are rescued by exogenous Rif1 expression [59]. In addition, telomere length heterogeneity, telomere loss, and telomeric DNA damage are observed in Rif1-depleted mESCs [56]. Collectively, Rif1, as a conserved regulator of DNA replication and repair, also maintains repetitive sequence silencing and protects them from DNA damage.

DNA topoisomerases (including topoisomerase I and II in mammals) can control the topological state of DNA by catalyzing the breaking and binding of DNA strands (Figure 1D). These enzymes are involved in DNA replication, transcription, chromatin condensation, and genomic stability [60]. Topoisomerase knockout in mice is embryonic-lethal [61]. DNA topoisomerase I changes the topological state during DNA replication by forming a break in ssDNA and re-ligation, whereas topoisomerase II creates a break in dsDNA and then re-ligates in an ATP-dependent manner. Recent studies have shown that DNA topoisomerases and their special inhibitors (e.g., CPT, VP-16) could form a covalent complex, and then mediate DNA cleavage on the purine–pyrimidine repeat sequence (e.g., AT-rich, GT-rich) or tandem repetitive sequences (e.g., telomeric repeat DNA, rDNA), which may form a DNA secondary structure (e.g., triplex DNA, cruciform structure) [62,63,64,65,66]. Treatment of mESCs and human ESCs (hESCs) with etoposide results in massive cell death. DNA isolated from etoposide-treated cells (4 h treatment and 20 h recovery) exhibited distinct DNA laddering, rather than a DNA smear, suggesting that topoisomerase II inhibitor induces the cleavage of telomeric DNA [67]. In addition, mapping studies have shown that cellular topoisomerase II cleavage sites are located near the DNase I-hypersensitive regions, such as heat shock locus, rRNA, and 28S rDNA [63,68,69]. DNase I-hypersensitive sites are associated with transcription activation and the accessibility of chromatin in mammalian cells [70]. Thus, these findings suggest a link between topoisomerase II site selection and chromatin structure.

## 4. Homologous Recombination (HR) Factors in Replication Fork Protection and Error-Free DNA Repair

**Figure 2:**
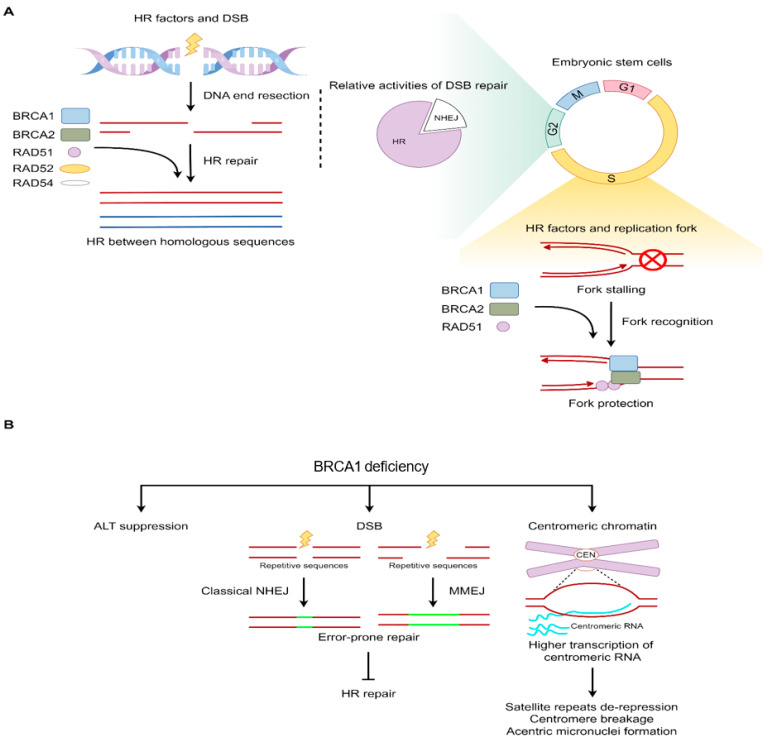
HR factors in replication fork protection and error-free DNA repair. (**A**) HR factors play a role in replication fork recognition and protection at S phase and error-free DNA repair at G2 phase in ESCs. The red and blue lines represent the sister chromatid. The curved red lines represent DNA replication. The incorrect symbol in the circle represents fork stalling. (**B**) BRCA1 deficiency indicates the inhibition of ALT events, the selection of DNA repair for repetitive sequences, satellite de-repression, and centromere instability. The red double line with a gap represents damaged DNA. The green lines represent repaired DNA. The curved blue lines represent centromeric RNA.

Compared to fibroblasts, ESCs exhibit distinct cell cycle characteristics, including a shorter G1 phase, which results in an overall longer percentage of S phase [49,51]. The shorter G1 phase minimizes the induction of differentiation stimulation signals, thereby helping ESCs to maintain pluripotency, which may be beneficial for embryonic development [71]. Due to the lack of G1 checkpoints in ESCs, cells damaged by exogenous stress are not arrested at the G1-phase checkpoint, but proceed to the S phase [72,73]. The longer S phase in ESCs utilizes homologous recombination (HR) factors to prevent DNA breaks and cell death through a replication-coupled pathway, particularly in regions containing repetitive DNA sequences [74] (Figure 2A). The high levels of HR factors are exhibited in ESCs throughout the cell cycle, e.g., RAD51, RAD52, RAD54, BRCA1, and BRCA2 [72,74,75,76]. HR factors inhibit replication fork collapse during the S phase to promote effective replication fork progression [77,78,79]. In addition, stalled replication forks can be stabilized and restarted by HR factors (Figure 2A). At the late S phase and early G2 phase, when DNA double-strand break (DSB) occurs, ESCs predominantly utilize error-free HR repair, rather than error-prone non-homologous recombination (NHEJ) [72,75,80] (Figure 2A). Therefore, specific cell cycle characteristics and more effective HR repair mechanisms enable ESCs to effectively tolerate various stresses and maintain genomic integrity.

For repetitive sequences, HR facilitates the replication efficiency of G-rich telomeric repeats. When telomerase is not yet activated at the early stages of embryonic development, telomere extension and maintenance mainly rely on HR, also known as the ALT (alternative lengthening of telomeres) mechanism [81,82]. Recent studies have found that ALT-related factor Dcaf11 plays an important role in telomere stability in early mouse embryos by activating Zscan4 and promoting HR in two-cell-like cells (2CLCs) [83]. Dcaf11 deficiency can lead to telomere shortening, which, in turn, leads to a significant decrease in the hematopoietic reconstitution ability in Dcaf11 knockout mice [83]. Moreover, cells lacking HR exhibit a marked reduction in telomere replication efficiency, and the suppression of ALT events has been observed in BRCA1/2-deficient cells [84,85] (Figure 2B). Additionally, Murine Brca1 deficiency in mESCs has also been observed in the presence of telomere dysfunction [86], supporting the notion that telomere recombination reactions are a mechanism for ALT occurrence.

HR factors are pivotal in selecting the DNA damage repair pathway for embryonic development. For example, BRCA1 is critical for DSB repair via HR. BRCA1 is upregulated both in human male and female germ cells and in preimplantation embryos [87]. Brca1-deficient mice are embryonic-lethal, characterized by neuroepithelial abnormalities [88]. Previous studies have shown that 53BP1 loss can partially rescue embryonic lethality in BRCA1 knockout mice, but not HR repair [89], suggesting that BRCA1-mediated HR repair is important in maintaining genome integrity. In addition, Brca1-deficient ESCs displayed a decreased error-free HR frequency and an increased frequency of NHEJ, which was corrected by the expression of a Brca1 transgene [90,91] (Figure 2B). In general, human BRCA1 mutation carriers are heterozygous, as the BRCA1 homozygotic mutation is embryonic-lethal [92]. As a tumor suppressor gene, BRCA1 mutations significantly increase the risk of breast, ovarian, prostate, and other cancers in carriers [93,94]. Furthermore, a recent study has shown that the heterozygous Brca1 mutation initiates mouse genome instability at the embryonic stage [95], suggesting the role of Brca1 in genome stability, early embryonic development, and carcinogenesis.

Whole-genome sequencing of DNA samples from heterozygous Brca1 mice revealed genome instability at the embryonic stage with dynamic changes [95]. Repetitive sequences, including simple repeat and retrotransposons (LINE, SINE, and ERV), are vulnerably attacked [95] (Figure 2B). Increased microhomology-mediated end joining (MMEJ) and single-strand annealing (SSA) are observed in Brca1-mutant mice. Furthermore, Brca1 also binds to satellite DNA regions and maintains heterochromatin-mediated transcriptional silencing. Brca1 deficiency in mice resulted in transcriptional de-repression of the tandemly repeated satellite DNA, accompanied by a reduction in condensed DNA regions in the genome [96] (Figure 2B). Specifically, BRCA1-deficient cells show impaired localization of CENP-A, increased transcription of centromeric RNA, heightened breakage at centromeres, and the formation of acentric micronuclei [97]. These findings underscore the critical role of BRCA1 as an HR factor in the regulation of DNA repair pathway choice, and demonstrate that effective and accurate HR repair in ESCs maintain genome integrity.

## 5. High Mismatch Repair (MMR) Inhibits Recombination between Diverged Sequences

**Figure 3:**
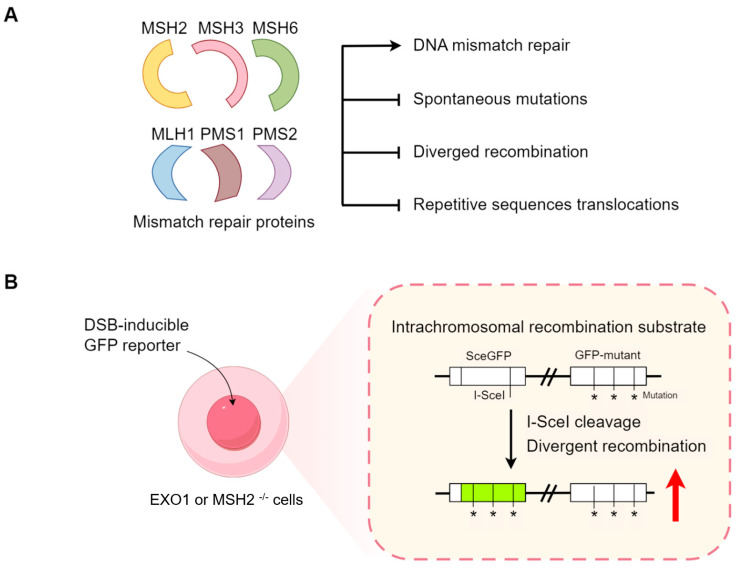
MMR inhibits recombination between diverged sequences. (**A**) MMR proteins play an important role in inhibiting spontaneous mutation, diverged recombination and repetitive sequence translocation. Different colors and shapes represent MMR proteins. (**B**) EXO1 or MSH2 inhibits recombination between diverged sequences, rather than recombination between identical, homologous sequences. The curved arrow represents the transfection of the reported plasmid into the cells. The black asterisks (*) represent GFP-mutant sites. The green box represents GFP expression. The red arrow represents increased GFP expression level.

Although multiple DNA damage response (DDR) mechanisms are present in all cell types, different cells are exposed to different types of damage based on their location and function in the body, leading to the specificity of damage repair pathways [98]. For example, rapidly proliferating cells, such as ESCs, are more prone to DNA replication mismatch and exhibit heightened mismatch repair (MMR) activity [99,100]. MMR is a highly conserved DNA repair mechanism that mainly utilizes mismatch repair proteins (e.g., MLH1, MSH2, MSH3, MSH6, PMS1, PMS2) to repair base–base mismatches and insertion/deletion mispairing, which is generated during DNA replication and recombination, thus maintaining genomic stability [72]. Microsatellite (MS) refers to repetitive sequences in series with a few nucleotides (mostly 1–6) in the genome. In fact, microsatellites are inherently unstable during DNA replication, which means that the length of the microsatellite sequences may vary. MMR deficiency causes mutation accumulation and repetitive sequence insertions or deletions, resulting in microsatellite instability (MSI) [101,102]. Thus, MMR protects genome stability from the mutation accumulation generated by the insertion/deletion mispairing of repetitive sequences (Figure 3A).

MMR also plays an essential role in ESCs. Studies using cells transfected with MMR reporter plasmids showed that MMR repair capacity is low in MEFs, but highly active in ESCs. As expected, the repair level of ESCs with MMR defects was several times lower than wild-type ESCs. These findings ascertain the role of MMR in ESCs and explain why ESCs do not accumulate more mutations after multiple passages [103,104]. MMR proteins also determine whether cells follow the DNA repair pathway or undergo the fate of apoptosis. Interestingly, high levels of endogenous MSH2 protein in hESCs promote cell apoptosis, while low levels promote DNA repair, as occurs in differentiated cells [103,105]. Compared with MSH2 knockout hESCs, even a low level of MSH2 protein can still reduce the number of spontaneous mutations in hESCs, suggesting that MMR plays an important role in regulating the level of mutagenesis in preimplantation embryonic cells. Furthermore, for microsatellite stability, the low-grade MSI mediated by MMR proteins may be characteristic of embryonic stem cells (Figure 3A).

In addition, MMR deficiency is related to frameshift mutations or loss of function in coding genes (*TGF-β RII*, *IGFIIR*, *BAX*, *P53*) in cancer. It was reported that there were two hotspot sites in the type II transforming growth factor-β receptor (TGF-β RII) gene in MMR-deficient tumor cells from patients with sporadic colorectal cancer: one fell within the 6 bp GT dinucleotide repeat and the other fell within an (A)_10_ mononucleotide repeat, resulting in the generation of TGF-β RII truncations [106]. Accordingly, MMR deficiency may be involved in carcinogenesis.

MMR proteins are crucial for preventing abnormal recombination between non-identical repetitive sequences (Figure 3). It is known that using an identical sequence for the HR pathway is robust, while recombination between diverged or non-identical sequences is suppressed in many organisms, such as ALU repeats in humans [107,108] (Figure 3B). It was found that up to 1.4% of divergent sequences led to a significant reduction in recombination between diverged sequences in mESCs [102,109]. In this context, MMR proteins inhibited abnormal recombination between diverged sequences. Exonuclease I (EXO1), as a canonical MMR protein, suppressed recombination between diverged sequences in mammalian cells, but not recombination between homologous sequences [110]. When DSB-inducible GFP reporters were introduced into Exo1^−/−^ mESCs, the recombination repair pathway between diverged sequences was significantly increased, whereas the recombination between homologous sequences was not affected [110] (Figure 3B). In Msh2^−/−^ cells, similar results were also observed [102,109] (Figure 3B). These results suggest that the failure to suppress recombination between diverged sequences impairs genome stability, potentially leading to tumorigenesis and chromosomal structural changes, such as diverged repetitive sequence translocations.

## 6. LINE-1 Retrotransposon Drives DNA Damage and Transposition

**Figure 4:**
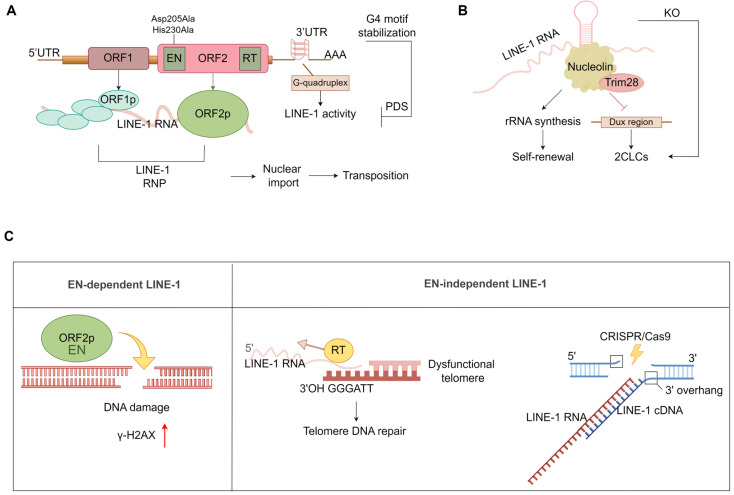
LINE-1 retrotransposon drives DNA damage and transposition. (**A**) The structure of human LINE1 element. LINE-1 is constituted by a 5′ UTR with promoter, ORF1, ORF2, and a 3′ UTR with a poly(A) tail. ORF1 encodes RNA binding protein ORF1p (arrow shown), and ORF2 encodes a protein ORF2p (arrow shown), which has both endonuclease (EN) and reverse transcriptase (RT) activity. In the 3′ UTR of LINE-1 elements, G-rich sequences are the hallmark of young LINE-1 retrotransposon. ORF1p, ORF2p, and G-quadruplex structures contribute to its transposition. (**B**) LINE-1 RNA recruits Nucleolin/Trim28 to repress the 2-cell marker Dux and activate rRNA synthesis. LINE-1 knockdown in murine zygotes arrests preimplantation development at the two-cell and four-cell stages. (**C**) LINE1-encoded endonuclease cleaves genomic DNA and increases γH2AX levels. LINE-1 EN mutants use endogenous exposure 3′ OH GGGATT overhang as a primer to insert into dysfunctional telomeres. DSB sites induced by CRISPR/Cas9 promote de novo LINE-1 insertion, which is EN-independent and RT-dependent. The red arrow represents increased γH2AX level.

LINE-1, a type of non-LTR retrotransposon, comprises approximately 21% of the human genome and is known for its ability to spontaneously transpose. LINE-1 is constituted by a 5′ UTR with promoter, ORF1, ORF2, and a 3′ UTR with a poly(A) tail. ORF1 encodes a 40kDa RNA binding protein ORF1p, and ORF2 encodes a 150 kDa protein ORF2p, which has both endonuclease (EN) and reverse transcriptase (RT) activity [111,112] (Figure 4A). ORF1p and ORF2p can bind RNA transcribed from LINE-1 elements (LINE-1 RNA) to form ribonucleoprotein particles (RNP) and then import to the nucleus to cause frequent transposition [113,114]. In the 3′ UTR of LINE-1 elements, G-rich sequences are the hallmark of young LINE-1 retrotransposon, and G-quadruplex structure formation by G-rich sequences contributes to its transposition. Interestingly, the stabilization of the G4 motif mediated by Pyridostatin (PDS) decreases LINE-1 activity [115].

Retrotransposons are dynamic and exhibit stage-specific activity, being active in early embryos, germlines, and pluripotent stem cells, while being inactive in somatic cells. However, recent research has demonstrated that LINE-1 is also active in human brain tissue and tumors [116,117,118]. Despite their abundancy, LINE-1s have undergone mutations or fragment loss, resulting in the loss of transposable ability. It is estimated that only about 100–150 LINE-1s retain active transposable ability [119,120].

LINE-1s are also crucial for early development. LINE-1 knockdown resulted in reduced RT activity, inhibited mESC self-renewal, and induced 2CLC conversion [121] (Figure 4B). LINE-1 RNA recruits Nucleolin/Trim28 to repress the two-cell marker Dux and activate rRNA synthesis [121]. In murine zygotes, LINE-1 knockdown irreversibly arrests preimplantation development at the two-cell and four-cell stages, which is consistent with the phenotype of the overall RT inactivation (excluding telomerase) in arrested embryos [121,122]. Although there was no direct evidence to confirm the role of LINE-1 elements that depend on its LINE-1 RNA (cis regulation) or LINE-1 ORF1/2 enzyme activity (trans regulation), the reported studies suggested that LINE-1 had trans- and strong cis-regulatory roles during early development to regulate gene expression and ensure evolutionary success.

Beyond its role in embryonic development regulation, LINE-1 can drive DNA damage, frequent transposition, and genetic variation and genome reorganization related to cancer, aging, autoimmunity, and neurodegeneration [114,116,120,123,124,125,126,127,128]. The endonuclease activity of ORF2p can cleave genomic DNA, with DNA double-strand breakage serving as the prerequisite for transposition [111] (Figure 4C). The overexpression of LINE-1 increased γH2AX (phosphorylated H2AX as DSB signal) levels during hESC differentiation into hippocampal neurons via neural precursor (NPC), while it was absent for EN- and RT-deficient LINE-1, suggesting LINE-1 drives DSB damage dependent on its enzyme activity [129].

Interestingly, LINE-1 can also facilitate transposition without relying on its endonuclease activity (Figure 4C). In both dysfunctional telomeric and non-homologous end joining (NHEJ) defective (e.g., DNA-PKcs or XRCC4 deficient) cells, approximately 30% of EN-independent LINE-1 used endogenous exposure 3′ OH GGGATT overhang as a primer to insert into dysfunctional telomere [130,131], suggesting that LINE-1 activation at the two-cell stage (without telomerase activity) may contribute to the maintenance of telomere length. Thus, under certain conditions, EN-independent retrotransposons could represent a telomerase-independent pathway of telomere maintenance. Considering the telomere maintenance in *Drosophila* (telomerase deficiency), the telomere length is elongated by active retrotransposons, which belong to non-LTR retrotransposons [132]. Moreover, the retrotransposon R2 also maintains ribosomal DNA (rDNA) repeat maintenance in *Drosophila* [133]. Collectively, non-LTR retrotransposons may be an ancestral mechanism of repetitive sequence maintenance.

In addition, DSB sites induced by CRISPR/Cas9 promote de novo LINE-1 insertion in Hela and HEK293T cells, which frequently occurs in an EN-independent and RT-dependent manner (Figure 4C). However, such events are rare during genome editing by prime editors (PE), cytidine, or adenine base editors (CBE or ABE), consistent with the reduced DSB [134]. It is reported that over 2500 de novo LINE-1 insertions occurred at multiple CRISPR/Cas9 editing sites in HEK293T, HeLa, and U2OS cells [134]. These results demonstrate that the efficiency of EN-independent LINE-1 retrotransposon insertion is related to the degree of DNA damage. Based on LINEs belonging to the non-LTR retrotransposon, in the future, EN-independent LINE-1s or non-LTR retrotransposons combined with CRISPR/Cas9 technology may be used as a reprogrammable RNA-based gene insertion tool. This may cause genomic instability, as well as new ways of DNA damage repair and the formation of new genes. However, the effects and safety of retrotransposon insertion in hESCs and iPSCs that are frequently used for CRISPR/Cas9 editing remain unclear and need further exploration.

Furthermore, DSB repair is an important trigger for the recombination of retrotransposon elements. During iPSCs’ differentiation into neurons, non-allelic homologous recombination (NAHR) between ALU and LINE-1 exhibits a significant increase in neurons compared with iPSCs [135]. A higher rate of young AluY and LINE-1Hs NAHR in brain samples is found compared with kidney and liver samples. Through exploring the hotspots of retroelement NAHR, somatic intra-chromosomal NAHR is enriched in human centromeres (within or in the proximity of centromeres). Expanding to the relationship of NAHR and sporadic Parkinson’s disease (PD), a significantly higher count of NAHR events was from ALU and LINE-1 (respective averages of 84% and 14%), while a minor contribution of other repeats to NAHR were observed in the human genome (2%) [135], suggesting a link between retrotransposon element recombination and genomic instability in neurodegeneration.

## 7. Dynamic Chromatin Structure in Response to DNA Damage

In the early stages of embryonic development, cells are highly sensitive to DNA damage caused by chemicals, radiation, or viruses. For instance, certain chemicals can cause 100% of hESCs to die within 5 h, whereas other types of cells may require up to 24 h. Compared to differentiated cells, early embryonic development and pluripotent stem cells, which possess more relaxed chromatin structures [136,137], are more susceptible to DNA damage and death. It is well documented that there are dynamic changes in chromatin structure during early embryonic development, characterized by a gradual decrease in chromatin accessibility. This process ultimately leads to the establishment of a more organized chromatin structure through mechanisms such as chromatin loop extrusion, topologically associated domains (TADs), and chromatin compartments (A and B), all of which are related to DNA damage repair pathway choice.

Euchromatin, being less condensed, is more prone to DNA damage compared to heterochromatin. For efficient DNA damage repair, chromatin relaxation is a prerequisite for DNA repair machineries to access DSBs. This relaxation often involves a temporary increase in the acetylation levels of core histones, which occurs in a dose- and time-dependent manner following DNA damage [138]. Chromatin compartments are correlated with the observed transcriptional activity, containing relatively active (A) and inactive (B) regions. During hESC-derived lineage specification, approximately 36% of A/B compartment switching was observed through Hi-C interaction maps analysis [139]. Notably, there appears to be a large expansion of the B compartment upon differentiation of hESCs to mesenchymal stem cells (MSCs), accompanied by relatively subtle corresponding changes in gene expression [139]. The changes in the A/B compartment correspond to a single or multiple TADs, suggesting that TADs may be the units of dynamic changes in chromatin compartments.

**Figure 5:**
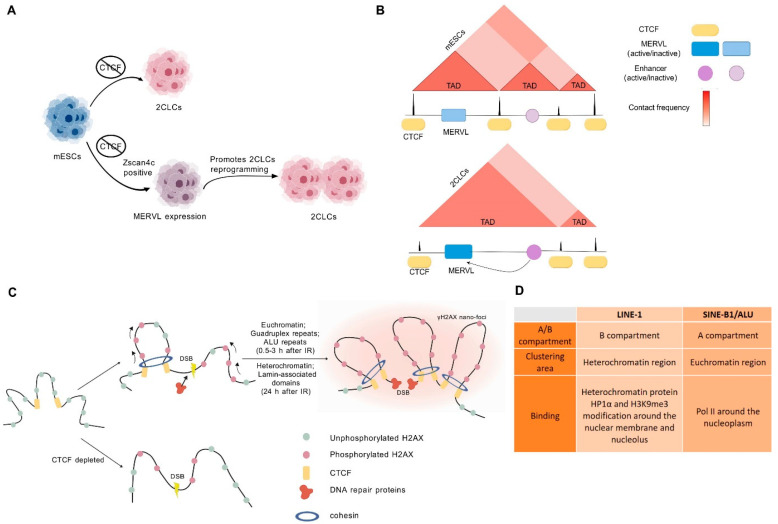
Dynamic chromatin structure in response to DNA damage. (**A**,**B**) CTCF depletion leads to spontaneous 2CLC reprogramming from mESCs. In addition, overexpression of Zscan4c in CTCF-deficient ESC increased 2CLC conversion, and Zscan4c may activate MERVL to promote 2CLC reprogramming in the context of weakened CTCF-induced TADs. (**C**) CTCF and cohesin establish γH2AX foci and nano-foci formation, and recruit DNA damage proteins to ensure proper DSB repair. Once CTCF depletion causes the loss of higher-order chromatin structures, it impairs bidirectional spread of γH2AX foci (double arrows shown) and DNA repair (one arrow shown) within TADs. (**D**) The distribution of SINE-B1/ALU and LINE-1 repeat sequences is correlated with the A/B compartment. LINE-1 is located in the B compartment, predominantly within the heterochromatin region, where heterochromatin protein HP1α and H3K9me3 modification bind around the nuclear membrane and nucleolus. SINE-B1/ALU aggregates in the A compartment, situated in the euchromatin region, where Pol II binds around the nucleoplasm.

It is known that CCCTC-binding factor (CTCF) is involved in chromatin loop extrusion and the insulation of TADs [140]. An increase in CTCF levels is necessary for the gradual establishment of TADs during human embryonic development [141]. CTCF is also crucial for mouse embryonic development, and the depletion of CTCF leads to spontaneous 2CLC reprogramming from mESCs [142], suggesting that the dynamics of TADs are crucial for early embryonic development and totipotent-like cell reprogramming (Figure 5A,B). In addition, the overexpression of Zscan4c in CTCF-deficient ESC increased the conversion to 2CLCs, and Zscan4c may activate MERVL to promote 2CLC reprogramming in the context of weakened CTCF-induced TADs [142], suggesting that TADs are essential for cell fate determination during early embryonic development (Figure 5B).

CTCF, a highly conserved zinc finger protein, dynamically binds to genomic DNA during the early stages of embryonic development in a DNA methylation-sensitive manner, influenced by its RNA binding activity or regulated protein, such as ADNP [143,144,145,146]. Chromatin immunoprecipitation and sequencing (ChIP-seq) analysis have shown that CTCF binds to repetitive sequences, such as rDNA, telomeres, sub-telomeres, and transposable elements, which is likely related to the chromatin structure at these locations [146,147,148,149,150]. It has been reported that CTCF and cohesin are integral components of most human sub-telomeres, regulating TERRA transcription and stabilizing telomere-end capping. The depletion of CTCF or cohesin subunit Rad21 causes telomere-induced DNA damage foci (TIF) formation, and destabilizes TRF1 and TRF2 binding to the TTAGGG proximal sub-telomere DNA, suggesting its important role in telomere-end protection [148]. Although not all CTCF sites form loops and insulate TADs, CTCF still plays an important role in preventing DNA damage and facilitating subsequent damage repair through its genomic binding.

Recent studies have indicated that TADs are functional units of the DNA damage response [151]. On the one hand, the genomic-wide weakening of TADs, mediated by CTCF depletion during 2CLC reprogramming, is accompanied by increased γH2AX levels and endogenous MERVL transcription levels [142]. A recent study reported that novel chromatin loops are enriched for chromatin accessibility, overlapping potential CTCF binding sites, along with the aberrant expression of repetitive sequences including ERVs and satellite classes [152]. On the other hand, CTCF can rapidly localize to DSB sites upon DNA damage, potentially promoting HR repair to prevent genomic instability. CTCF and cohesin established γH2AX foci and nano-foci formation, recruiting DNA damage proteins, such as ATM and BRCA2, to ensure proper DSB repair kinetics [153,154,155,156]. CTCF depletion impaired the spread of γH2AX nano-foci and DNA repair [157,158], suggesting its regulatory role in the structural component of the DNA damage response. These results demonstrate that TADs mediated by CTCF are involved in DNA damage repair, and are crucial for protecting genomic integrity (Figure 5C). During DNA damage and DSB repair, a trend was observed from euchromatin-to-heterochromatin repair, related to the chromatin compaction state. Euchromatin, quadruplex repeats, and ALU repeats are repaired earlier than heterochromatin and lamina-associated domains [157]. Mouse heterochromatin contains non-coding major satellite repeat DNA elements, and satellite repeat transcripts modulate heterochromatin condensates in mESCs [159,160]. On CRISPR-Cas9-mediated DSB induction at major satellites’ DNA, heterochromatin was dramatically decondensed, along with increased γH2AX levels [157]. In addition, the stabilization of TADs safeguards the genome integrity in response to DSB. A recent study reported that 53BP1 accumulates at compact TADs of DSB sites, and then RIF1 is recruited to the TAD boundary and 53BP1 and RIF1 stabilize TAD structures into a circular arrangement only 10–15 min after DSB generation [57]. The topological arrangement of DSB-flanking chromatin is independent of DNA repair [57]. These results demonstrate that the maintenance of TAD structures after DSB is beneficial for genome integrity and may regulate the expression of repetitive sequences.

The distribution of SINE-B1/ALU and LINE-1 repeat sequences correlates with A/B compartments in mice and humans [161]. LINE-1s are located in the B compartment, predominantly within the heterochromatin region, where heterochromatin protein HP1α and H3K9me3 modification bind around the nuclear membrane and nucleolus. SINE-B1/ALU aggregates in the A compartment, situated in the euchromatin region, where Pol II binds around the nucleoplasm (Figure 5D). The spatial segregation of LINE-1 and SINE-B1/ALU-rich compartments within the nucleus is a conserved feature [161]. Furthermore, LINE-1 RNA binds its repeat sequences and promotes the spatial segregation of LINE-1 in B compartments. It also specifically promotes the phase separation of HP1α [161]. The depletion of LINE-1 RNA through the microinjection of antisense oligonucleotides (ASO) significantly decreases the Hi-C segregation indexes of LINE-1 in B compartments compared to control mESCs, underscoring the critical role of LINE-1 RNA in mediating spatial segregation and phase separation to maintain the 3D chromatin structure. Similarly, major satellite repeat RNA contributes to the constitutive heterochromatin dependent on the phase separation of HP1ɑ [162]; MERVL triggers the changes in chromatin organization during the reprogramming of mESCs to 2CLCs [163]; and HERV-H demarcates TADs in hESCs [164]. These finding imply that repetitive sequences and their transcripts are pivotal in maintaining the biophysical state of chromatin architecture and ensuring chromosome stability in ESCs. Although the response of LINE-1 and SINE-B1/ALU-rich compartments to DNA damage is still unclear, the phase separation and spatial separation in chromatin compartments are likely to maintain genomic stability.

In addition, a recent study shows that DSB drives the formation of a new chromatin compartment (termed the D compartment) in cancer cells through the clustering of damaged TADs [151]. This process relies on polymer-phase separation such as γH2AX and 53BP1. D chromatin has dual effects, which not only protect genome stability, but also may promote translocation and drive carcinogenesis [151]. Collectively, the regulation of chromatin compartments may be important for early embryonic development, carcinogenesis, and the transformation in cell fate. Therefore, investigations into the role and molecular mechanism of repetitive sequences and their RNA transcripts in TADs and chromatin compartments will provide valuable insights into how genome folding and chromatin structure respond to DNA damage. This enhanced understanding will deepen our knowledge of these complex biological processes, shedding light on the intricate interplay between genomic architecture and cellular responses to exogenous challenges.

## 8. Conclusions and Perspectives

Most repetitive sequences in the human genome originate from parasitic sequences such as viruses and transposable elements [14]. To defend against the adverse effects of these parasitic sequences, hosts have developed mechanisms, such as RNA interference, epigenetic and chromatin regulations, RNA modifications, and DNA methylation, to regulate their expression levels, transposition, and stability [165]. During early embryonic development, the expression of retrotransposons is dynamic and stage-specific. The expression of most retrotransposons is inhibited before implantation, while retrotransposons are activated during zygotic activation, such as LINE-1 and MERVL [121,163,166,167]. On the contrary, in 2CLC conversion from mESCs, MERVL is specially activated [26,168,169]. In fact, about 100–150 LINE-1 elements can integrate into the genome through a “copy and paste” mechanism, producing disperse copies throughout the genome [120]. It has been shown that retrotransposon elements are not randomly distributed in the genome [170], but rather are primarily inserted into introns and intergenic regions, with little in encoded exons, and no insertion in some genes during genome evolution [171,172]. While retroviruses become endogenized and have the potential to retain cis- and trans-regulatory activity under epigenetic control in ESCs, it is unknown how retrotransposon expression levels and their transposition are dynamically regulated. Although the arms race between parasitic sequences and hosts is the main driving force of genome evolution, the mutualist mechanism during early embryonic development remains elusive. Exploring the stability and function of repetitive sequences could provide valuable insights into cell diversity, embryonic cell development, and disease.

It is well established that the human immune system not only protects us from exogenous viral infection, but also constantly prevents the resurgence of endogenous retroviruses [173,174,175,176]. During early embryonic development, pluripotent stem cells, not somatic cells, can be resistant to certain viral infections (including exogenous and endogenous viruses) through RNA interference response mechanisms [177], such as those mediated by DICER. In mESCs, the type III ribonuclease Dicer detects and cleaves dsRNA substrates to produce small interfering RNAs (siRNAs), with dsRNA mainly derived from the retrotransposon, thereby regulating retrotransposon levels and infection [178,179,180]. In addition, in response to exogenous and endogenous viral infection, ESCs do not depend on type I interferons to induce the expression of interferon-stimulated genes (ISGs), whereas constitutive ISGs do [181,182]. Recent studies have shown that ERVs activate ISGs in mouse mesothelioma development [183]. It is still unclear why ESCs can express high levels of ISGs in the absence of infection and whether active ERVs play a role in this process. Therefore, pluripotent stem cells, as a general property of interferon-independent antiviral resistance, have become a good model for studying antiviral innate immunity [184]. In addition, during species evolution, endogenous retroviruses play an important role in the evolution of mammalian genomes and the emergence of new phenotypes. A typical example is that the formation of the placenta, an important organ supporting mammalian embryonic development, is related to endogenous retroviruses, which can promote placental development by encoding viral proteins or providing tissue-specific transcriptional regulatory elements (such as enhancers or promoters) [185,186]. The further exploration of antiviral resistance mechanisms in pluripotent stem cells will enhance the understanding of evolutionary mutualism mechanisms and be beneficial to human health.

Autonomous retrotransposons are the major source of endogenous DNA damage and subsequent transposition. Focusing on telomeric DNA and rDNA in *Drosophila*, retrotransposons can transposition, and may be an ancient mechanism of repetitive sequence maintenance [130,131,132,133]. In addition, DSB sites induced by CRISPR/Cas9 promote de novo LINE-1 insertion, rather than reduced DSB sites induced by PE, CBE, or ABE, indicating that the number of DSB sites maybe a key factor in transposition [134]. The DSB sites mediated by CRISPR/Cas9 prioritize NHEJ repair, and the correlation between transposition and DNA repair remains unclear. Thus, further extensive research is necessary to elucidate the role of autonomous retrotransposons in DNA damage repair.

Given the capacity of ESCs to self-renew and differentiate into somatic cells, the dynamic chromatin structure is associated with cell fate decisions, such as during iPSC reprogramming. It is reported that SINE-B1/ALU distributions are correlated with the A compartment, whereas LINE-1 correlates with the B compartment [161]. However, the regulatory role of repetitive sequences in the dynamics of the A/B department during stem cell fate transition remains unclear. Future research on repetitive sequences will need to develop new methods and multi omics analysis to determine genomic regions based on cellular status and to ascertain which repetitive sequences are associated with these regions. It will be crucial to determine the molecular mechanisms on how the organization of the genome around the nucleus is established. In addition, the studies have shown that repetitive sequences not only act on the function of promoter, enhancer, and insulator, but also contribute to TAD boundaries [149], such as SINEs [187,188]. How SINEs recognize chromatin boundary positions and insertions, and whether SINEs serve as a marker to indicate chromatin folding, still need extensive exploration. In the future, by influencing the organization of regulatory domains in mammalian genomes, it is conceivable that the new TE insertion can independently alter gene regulation and influence embryonic stem cell fate.

Repetitive sequences play an important role in early embryonic development and disease progression, influencing gene expression, transcriptional regulation, and chromatin structure through trans- and cis-regulatory roles. Based on current research progress, we think that the stability and function of repetitive sequences is the main research direction, as well as understanding the relationship between embryonic development and diseases.

## Data Availability

Not applicable.
